# Detecting substrate glycans of fucosyltransferases with fluorophore-conjugated fucose and methods for glycan electrophoresis

**DOI:** 10.1093/glycob/cwaa030

**Published:** 2020-05-02

**Authors:** Zhengliang L Wu, Mark Whittaker, James M Ertelt, Anthony D Person, Vassili Kalabokis

**Affiliations:** Bio-techne, R&D Systems, Inc., 614 McKinley Place N.E., Minneapolis, MN 55413, USA

**Keywords:** fucosylation, fucosyltransferase, glycan analysis, glycosylation, Lewis X

## Abstract

Like sialylation, fucose usually locates at the nonreducing ends of various glycans on glycoproteins and constitutes important glycan epitopes. Detecting the substrate glycans of fucosyltransferases is important for understanding how these glycan epitopes are regulated in response to different growth conditions and external stimuli. Here we report the detection of these glycans on glycoproteins as well as in their free forms via enzymatic incorporation of fluorophore-conjugated fucose using FUT2, FUT6, FUT7, FUT8 and FUT9. Specifically, we describe the detection of the substrate glycans of these enzymes on fetal bovine fetuin, recombinant H1N1 viral neuraminidase and therapeutic antibodies. The detected glycans include complex and high-mannose N-glycans. By establishing a series of precursors for the synthesis of Lewis X and sialyl Lewis X structures, we not only provide convenient electrophoresis methods for studying glycosylation but also demonstrate the substrate specificities and some kinetic features of these enzymes. Our results support the notion that fucosyltransferases are key targets for regulating the synthesis of Lewis X and sialyl Lewis X structures.

## Introduction

Glycans are commonly found on cell membranes and secreted proteins. They are frequently terminated with sialic acids, negatively charged monosaccharides, and fucose, a deoxy hexasaccharide. For their unique physical properties, sialic acids and fucose are essential constituents of various glycan epitopes that are recognized by lectins and antibodies and play important biological roles (Becker and Lowe 2003; Sackstein 2009; Miyoshi et al. 2012; Schneider et al. 2017; Varki 2017).

Well-known fucosylated glycans include blood group H-antigen, Lewis structures and core fucosylated N-glycan. They are generated through various fucosyltransferases (Ma et al. 2006). H-antigen on red blood cells contains an α1,2-linked fucose introduced by FUT1 and FUT2 (Kelly et al. 1995). Lewis X (Le^x^) structure is a trisaccharide (Galβ1–4[Fucα1–3]GlcNAc) that has a fucose residue linked to a GlcNAc residue through an α1,3 linkage. Le^x^ structure can be sialylated at the Gal residue to become sialyl Lewis X structure (sLe^x^)(Neu5Acα2-3Galβ1–4[Fucα1–3]GlcNAc) that is the ligand for E-selectin and is essential for lymphocyte extravasation (Nelson et al. 1993). The α1,3-linked fucose on Le^x^ and sLe^x^ structures is introduced via several fucosyltransferases including FUT4, FUT6, FUT7 and FUT9 (Mondal et al. 2018). Among these enzymes, FUT7 is strictly active on sialyllactosamine (Sasaki et al. 1994), FUT4 and FUT9 are strictly active on lactosamine (Brito et al. 2008) and FUT6 is active on both structures (Weston et al. 1992). Lewis A (Le^a^) structure (Galβ1–3[Fucα1–4]GlcNAc) and its sialylated version sialyl Lewis A (sLe^a^) are isomers of Le^x^ and sLe^x^ structures and are fucosylated by FUT3 (Kukowska-Latallo et al. 1990). Core-6 fucosylation on the innermost GlcNAc of N-glycan introduced by FUT8 (Ihara et al. 2006) plays a critical role in the antibody-dependent cellular cytotoxicity (ADCC) of therapeutic antibodies (Jefferis 2009). For FUT8 substrate recognition, an unmodified β1,2-linked GlcNAc residue introduced to the α3 arm of N-glycan by MGAT1 (Kumar et al. 1990) is critical (Yang et al. 2017; Garcia-Garcia et al. 2020).

For their important biological roles, cellular display of fucosylated glycan epitopes (Adey et al. 2013) must be tightly regulated (Sackstein 2009). It is believed that this regulation is achieved via the establishment of precursor glycan pools and controlled expression of key FUTs. Upon environmental stimuli, cells can quickly convert the precursor glycans to functional epitopes via the action of these enzymes. Therefore, it is equally important to detect the glycan epitopes and their precursor glycans.

Previously, we described a direct fluorescent glycan labeling (DFGL) strategy to label and detect the substrate glycans of various sialyltransferases (Wu et al. 2019, 2020). In this report, we describe another DFGL strategy to label and detect the substrate glycans of some representative FUTs. More importantly, we describe electrophoresis-based methods for studying free glycans, which allows us to further analyze glycan composition and study kinetics of glycan epitope synthesis. The methods were demonstrated on several well-characterized glycoproteins, including fetal bovine fetuin that contains complex N-glycans and O-glycans (Ma et al. 2006), ribonuclease B that contains high-mannose N-glycans (Prien et al. 2009), insect cell expressed recombinant H1N1 neuraminidase that contains Man3 type oligo-mannose N-glycan (Wu et al. 2016) and Cantuzumab (Rodon et al. 2008) and the reference monoclonal antibody from National Institute of Standards and Technology (NIST mAb 8671) (Kashi et al. 2018) that contain complex N-glycans. By establishing a series of precursor glycans through enzymatic conversion, we revealed multiple intermediate products during the synthesis of Le^x^ and sLe^x^. Our results indicate that fucosylation is a much faster process than sialylation, suggesting that fucosylation is the step where the synthesis of Le^x^ and sLe^x^ is regulated.

## Results

### Detection of substrate glycans of α-2 and α-3 fucosyltransferases on fetal bovine fetuin

**Fig. 1:**
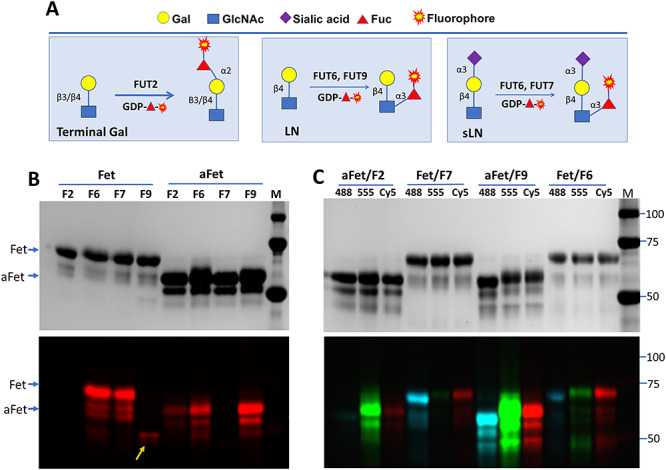
Probing substrate glycans of FUT2, FUT6, FUT7 and FUT9 on fetuin (Fet) and asialofetuin (aFet). (**A**) Strategies for probing substrate glycans of indicated FUTs. FUT2 recognizes both β3- and β4-linked terminal Gal. FUT9 recognizes terminal lactosamine (LN). FUT7 only recognizes sialyllactosamine (sLN). FUT6 recognizes both LN and sLN. Monosaccharide symbols follow the Symbol Nomenclature for Glycans (SNFG) system (PMID 26543186, Glycobiology 25: 1323–1324, 2015) details at NCBI. (**B**) Probing substrate glycans of FUT2(F2), FUT6(F6), FUT7(F7) and FUT9 (F9) on Fet or aFet with GDP-Cy5-fucose. FUT9 labeled itself (indicated by an arrow). (**C**) Probing substrate glycans of FUT2, FUT6, FUT7 and FUT9 on Fet or aFet with Alexa Fluor®555 (555), Alexa Fluor®488 (488) and Cy5-conjugated fucose. All reactions in (**B**) and (**C**) were incubated at 37^o^C for 30 min and then separated on SDS–PAGE and imaged with silver staining (upper panels) and fluorescent imager (lower panels). M, molecular marker.

To test whether we can detect the substrate glycans of α-2 and α-3 FUTs (Figure 1A), we first prepared Cy5-conjugated GDP-Cy5-Fuc and tested it as a donor substrate for FUT2, FUT6, FUT7 and FUT9 on fetal bovine fetuin and asialofetuin (Figure 1B). The labeled samples were then separated on sodium dodecyl sulfate–polyacrylamide gel electrophoresis (SDS–PAGE), followed by traditional protein gel redundant and fluorescent imaging. By comparing the images, it was found that these enzymes indeed can recognize Cy5-conjugated fucose (Cy5-Fuc) and transfer it to their substrate glycans. Specifically, we found that FUT2 and FUT9 can label asialofetuin, FUT7 can label fetuin and FUT6 can label both fetuin and asialofetuin. These results are consistent to the specificities of these enzymes reported in the literature (Weston et al. 1992; Sasaki et al. 1994; Brito et al. 2008; Mondal et al. 2018).

Next, we tested the tolerance of the FUTs for Cy5, Alexa Fluor® 488 and Alexa Fluor® 555 conjugated fucoses (Figure 1C). It was found that all three dyes were tolerated by the enzymes to different levels. FUT2 preferred Alexa Fluor® 555, FUT7 preferred Alexa Fluor® 488, FUT9 showed strong labeling with all three dyes and FUT6 showed consistent labeling with all three dyes with no obvious preference.

**Fig. 2:**
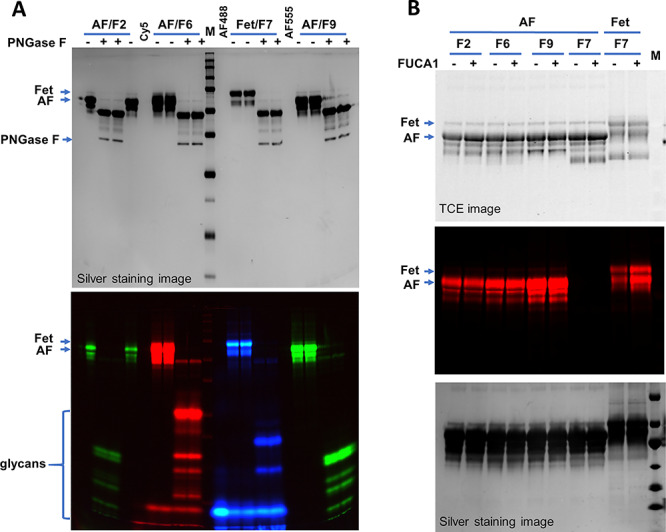
Characterizing the labeled glycans on fetuin (Fet) and asialofetuin (AF) with PNGase F and FUCA1. (**A**) PNGase F treatment of the labeled samples. AF was labeled by FUT2 (F2), FUT6 (F6) and FUT9(F9) with Alexa Fluor® 555 (green) or Cy5 (red). Fet was labeled by FUT7 (F7) with Alexa Fluor® 488 (blue). Labeled samples were then treated with PNGase F to release the glycans. (**B**) Effect of FUCA1 on the labeling. Samples without or with FUCA1 treatment were labeled by the indicated enzymes with Cy5. All samples were separated on 15% SDS–PAGE and imaged by silver staining, TCE staining and fluorescent imaging as indicated. M, western blot molecular marker.

To further understand the nature of the glycans labeled by these enzymes, the samples were either treated with PNGase F, an amidase that removes entire N-glycans from glycoproteins (Tarentino and Plummer 1994), or FUCA1, a lysosomal enzyme that hydrolyze α-fucose residues from glycans (Fukushima et al. 1985). When the FUT2-, FUT6-, FUT7- and FUT9-labeled samples were treated with PNGase F, all incorporated fluorescent signals were released (Figure 2A), suggesting that the substrate glycans for those enzymes on fetuin are exclusively carried by N-glycans. This experiment also demonstrated that free N-glycans can be separated well by gel electrophoresis. Among FUCA1-treated samples, 2-fold increase on labeling was observed on FUT7-labeled fetuin after the treatment, suggesting the preexistence of sialyl Lewis X on native fetuin sample.

### Probing fucosylation on therapeutic antibodies by FUT8 and FUT9

**Fig. 3:**
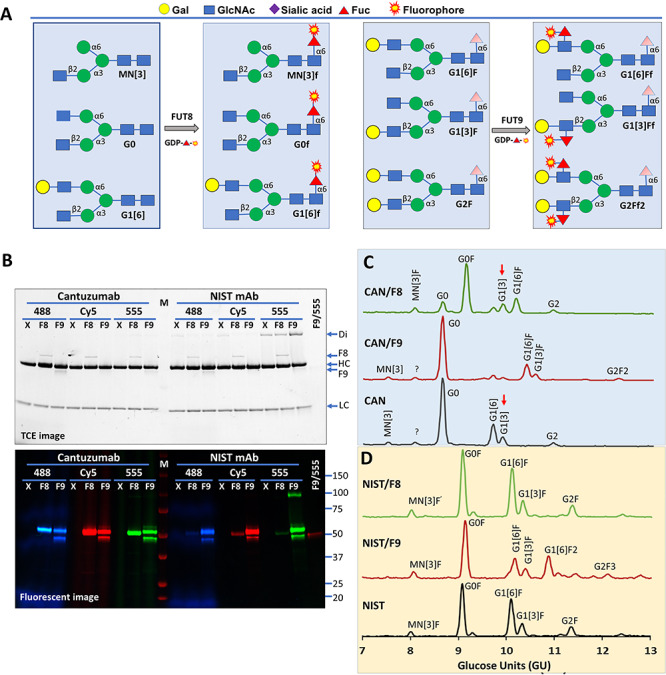
Probing fucosylation on Cantuzumab and NIST antibodies by FUT8 and FUT9. (**A**) Nomenclature of common antibody glycans and strategies for their detection. M, mannose; N, glucosamine; G, galactose; F, fucose; f, fluorophore-conjugated fucose. Only sugar residues at the nonreducing ends are reflected in the short names. Sugars in shade are flexible. (**B**) Probing substrate glycans of FUT8 (F8) and FUT9 (F9) on Cantuzumab, an anti-Muc1 therapeutic antibody, and NIST reference mAb 8671 using GDP-Alexa Fluor®555-fucose (555), GDP-Cy5-fucose (Cy5) and GDP-Alexa Fluor®488-Fucose (488) as the donor substrates. Cantuzumab was expressed in FUT8 knockout cells and is devoid of core-6 fucose. M, western molecular marker (Bio-Rad). All reactions were separated on 4–20% gradient SDS–PAGE and imaged with TCE staining (upper panel) and fluorescent imager (lower panel). FUT9 (at 50 kDa) exhibited self-labeling. X, no enzyme control; Di, unreduced dimer of the heavy chain; HC, heavy chain; LC, light chain. (**C**) GlyQ analysis of the Cantuzumab (CAN) after in vitro modification by FUT8 and FUT9, showing FUT8-modified G0 and G1[6] but not G1[3] (red arrows) and FUT9 modified G1[6] and G1[3]. (**D**) GlyQ analysis of the NIST mAb after in vitro modification by FUT8 and FUT9, showing that FUT8 had no modification on the glycans and FUT9 modified G1[6]F and G2F.

It is known that FUT8 can tolerate azido-fucose (Wu et al. 2016) and alkynyl fucose (Kizuka et al. 2016). Here we tested whether FUT8 can tolerate the three fluorophore-conjugated fucoses and detect its substrate glycans on therapeutic antibody Cantuzumab that lacks core-6 fucosylation and the standard reference antibody NIST mAb material 8671 (Figure 3). Both antibodies are IgGs that are known to contain an N-glycan site on their heavy chains (Reusch and Tejada 2015). To our expectation, significant amounts of Alexa Fluor® 555-, Alexa Fluor® 488- and Cy5-conjugated fucoses were introduced by FUT8 to Cantuzumab but not NIST mAb (Figure 3B). For comparison, the samples were also probed by FUT9, which showed comparable incorporation of the three dyes to both antibodies.

To provide evidence that the labeling was specific to the labeling enzymes, in a parallel experiment, glycans of in vitro fucosylated Cantuzumab and NIST mAb were analyzed on a Gly-Q™ Glycan Analysis System (Figure 3C and D). While FUT9 converted G1[6], G1[3] and G2 of Cantuzumab to G1[6]F, G1[3]F and G2F2, respectively, FUT8 converted M3N[3], G0 and G1[6] of the antibody to M3N[3]F, G0F and G1[6]F (Figure 3C), respectively, demonstrating the strict specificities of these two FUTs. It is noteworthy that the glycan G1[3] that contains a galactosylated β1,2-linked GlcNAc on its α3 arm was not modified by FUT8, confirming the notion that a free β1,2-linked GlcNAc on the α3 arm of N-glycan is critical for the substrate recognition by FUT8. Consistent to the labeling data in Figure 3B, G1[6]F and G2F on NIST mAb in Figure 3D were only modified by FUT9 but not by FUT8.

**Fig. 4:**
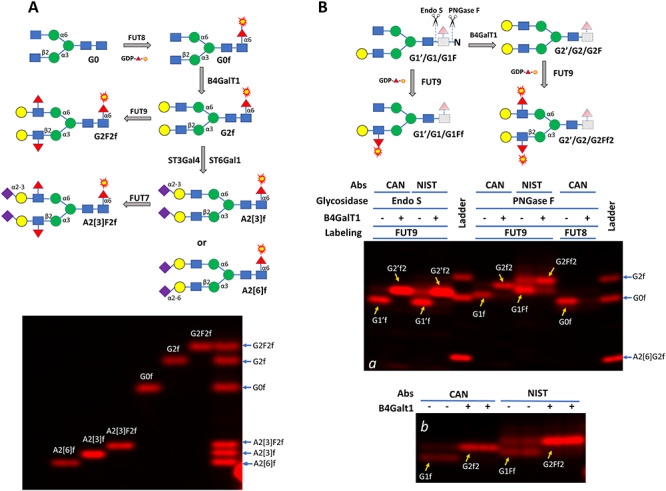
Establishing reference glycan standards (**A**) and characterizing glycans on Cantuzumab (CAN) and NIST mAb (NIST) (**B**). In (**A**), G0 was first labeled by FUT8 and then enzymatically converted to five other glycans, including G2F2 that carries Lewis X structure and A2[3]F2 that carries sialyl Lewis X structure. The labeled glycans were then separated on a 17% SDS–PAGE (about 0.25 ng each of the labeled glycan was loaded in each lane). In (**B**), glycans on Cantuzumab and NIST mAb (2.5 μg per sample) were released by either Endo S or PNGase F and then labeled by FUT9 or FUT8 directly or after galactosylation by B4GalT1. The labeled glycans were separated on a 17% SDS–PAGE together with some of the glycan standards generated in (**A**) (panel **a**). Endo S released glycans lack the core GlcNAc residue at the reducing ends (with dashed lines and light shades) and are indicated with prime symbols. For better viewing the glycan separation, labeling on PNGase F released glycans by FUT9 was repeated in panel **b**. Nomenclature follows the same rules of Figure 3. The galactose residue in the monogalactosylated glycans in (**B**) can be on either arm but only one is presented.

To further identify the substrate glycans for FUT8 and FUT9 on Cantuzumab and the NIST mAb, we established a glycan ladder via enzymatic conversion of FUT8-labeled G0 glycan (Figure 4A) and then compared the glycans released by Endo S and PNGase F from the two antibodies to the ladder (Figure 4B). Endo S is an endoglycosidase specific for the glycans on IgG, and its cleavage on IgG leaves the innermost GlcNAc residue of a target N-glycan attached to the protein backbone (Collin and Olsen 2001). One major PNGase F-released glycan from Cantuzumab matched G0 through FUT8 labeling, and the labeling was sensitive to B4GalT1 pretreatment, suggesting that the glycan is G0. FUT8 showed no labeling on Endo S-released glycans, as these glycans lacked glycosylation sites for FUT8 (data not shown). FUT9 labeling resulted one major lower band and one minor upper band on both Endo S and PNGase F-released glycans from both Cantuzumab and the NIST mAb, with the intensities of the two bands corresponding well with the peak intensities of G1 and G2 species in the GlyQ data of Figure 3C and Figure 3D. Moreover, the lower bands in Figure 4B were shifted to the upper bands by B4GalT1 treatment, suggesting that the two bands are corresponding to G1 and G2, respectively.

The above experiments not only demonstrated that the substrate glycans on antibodies can be labeled and detected but also demonstrated that glycans differ by one sugar residue such as G1’f, G0f and G1f in Figure 4B and even glycan isomers such as A2[6]f and A2[3]f in Figure 4A can be separated in SDS–PAGE.

### Detecting high-mannose glycans by FUT8

**Fig. 5:**
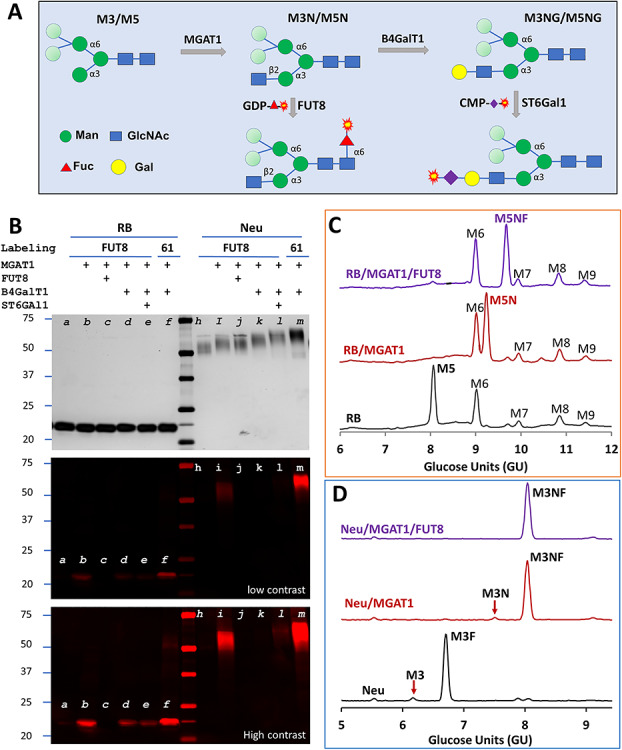
Detecting high-mannose glycans on glycoproteins by FUT8 and demonstration of the substrate specificity of FUT8. (**A**) Scheme for detecting high-mannose glycans. While the β1,2-linked GlcNAc introduced by MGAT1 on the α3 arm is essential for FUT8 recognition, the mannose residues in lighter shade on the α6 arm are flexible for substrate recognition. Further elongation of the α3 arm with B4GalT1 renders the glycan to be the substrate glycan for ST6Gal1. (**B**) Detecting Man5 on RNase B (RB) and Man3 on recombinant H1N1 neuraminidase monomer (Neu) and the substrate specificity of FUT8. Samples were pretreated with MGAT1, FUT8, B4GalT1 and ST6Gal1 with their respective unmodified donor substrates (indicated with +) before labeling. The pretreated samples were then labeled by FUT8 with GDP-Cy5-fucose or ST6Gal1 (61) with CMP-Cy5-Sialic acid. All samples were separated on SDS–PAGE and visualized by silver staining and fluorescent imaging. The middle and lower panels are the fluorescent image with different contrasts. Only MGAT1-modified sample was strongly labeled by FUT8, and further elongation reduced the labeling greatly. In parallel experiments, samples of RB (**C**) and Neu (**D**) were modified by MGAT1 or together with FUT8 with their natural donor substrates and then analyzed with GlyQ.

High-mannose glycans are related to serum clearance of therapeutic antibodies (Goetze et al. 2011) and are frequently targeted in broad neutralizing antibody responses during human immunodeficiency viral infection (Lavine et al. 2012). As such, detection of high-mannose glycans is particularly valuable. Here, we demonstrate a strategy to probe high-mannose glycans using FUT8 and show the substrate specificity of FUT8. Bovine ribonuclease B (RNase B) is known to contain high-mannose glycans (Prien et al. 2009). To test whether we can detect high-mannose glycans on a glycoprotein, a sample of RNase B was first treated with α1,3-mannosyl-glycoprotein 2-β-N-acetylglucosaminyltransferase (MGAT1) to introduce the α3 arm GlcNAc residue before labeling by FUT8 (Figure 5A). The addition of α3 arm GlcNAc residue by MGAT1 resulted in strong labeling by FUT8, and further galactosylation and sialylation significantly reduced the labeling (lanes at the left side of the marker in Figure 5B), which is consistent to the result in Figure 4B and the observation that G1[3] was not modified by FUT8 in Figure 3C. In addition, pretreatment of RNase B by FUT8 with unmodified fucose abolished the labeling completely, suggesting that the conjugation of Cy5 to fucose did not affect the substrate recognition by FUT8. As a positive control, an RNase B sample pretreated with MGAT1, and β-1,4-galactosyltransferase 1 (B4GalT1) was labeled with Cy5-conjugated sialic acid by ST6Gal1, which resulted in similar intensity of labeling (comparing lanes *b* and *f* in Figure 5B).

To identity the glycans labeled by FUT8 and ST6Gal1, sequentially modified RNase B samples were analyzed with Gly-Q™ Glycan Analysis System. The results indicated that only Man5 (M5) led to eventual modification by FUT8 (Figure 5C) and ST6Gal1 (Supplemental Figure 1). Since other high-mannose glycans including Man6, Man7, Man8 and Man9 can be converted to Man5 by α1,2-specific mannosidase (Avezov et al. 2008), in theory, all these glycans can be detected by FUT8 as well.

To test whether Man3 glycan can be labeled, monomeric Sf21 cell-expressed recombinant 1918 H1N1 influenza neuraminidase (Neu) that is known to contain both Man3 (M3) and core-6 fucosylated Man3 (M3F) (Wu et al. 2016) was tested by FUT8. Again, the sample was labeled significantly by FUT8 only after pretreatment with MGAT1, and the labeling was inhibited or abolished by additional pretreatment by B4GalT1 and ST6Gal1 (lanes at the right side of the marker in Figure 5B). Meanwhile, since the difference between Man5 and Man3 is on the α6 arms, these results also proved that the α6 arms are flexible for FUT8 recognition. In contrast to labeling on RNase B, the signal of FUT8 labeled Neu was only a fraction of that of ST6Gal1 labeled Neu (comparing lane *i* and *m* in Figure 5B). To understand this difference and confirm that Man3 was indeed modified, sequentially modified Neu samples were subject to GlyQ analysis. It was found that the precursor substrate glycan for FUT8 (M3) on Neu was only about 3% of that of the precursor substrate glycans for ST6Gal1 (both M3 and M3F) (Figure 5D), therefore explaining the difference on Neu samples labeled by FUT8 and ST6Gal1 in Figure 5B. Similar results were obtained when the experiment was repeated on both the monomeric and the dimeric recombinant 1918 H1N1 influenza neuraminidase prepared in different batches (Supplemental Figure 2).

### Study the enzymatic synthesis of Le^x^ and sLe^x^ epitopes using glycan gel electrophoresis

**Fig. 6:**
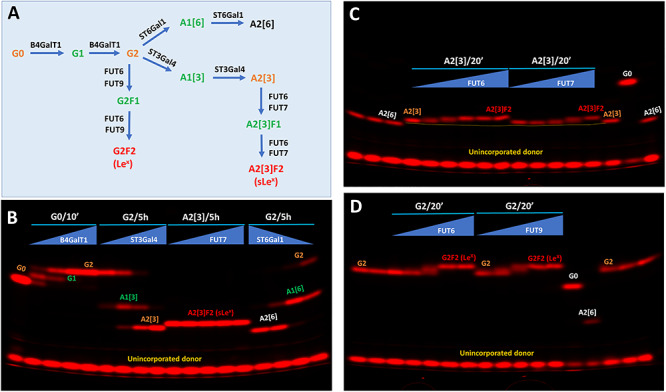
Kinetic comparison of fucosylation and sialylation on Lewis X (Le^x^) and sialyl Lewis X (sLe^x^) synthesis. (**A**) Schematic view of the enzymatic steps for Le^x^ and sLe^x^ synthesis on the antibody glycan G0. The glycan short names follow that of Figure 3. (**B**) Kinetic comparison of galactosylation on G0 by B4GalT1, sialyation on G2 by ST3Gal4 and ST6Gal1 and fucosylation on A2[3] by FUT7. The reaction time for B4GalT1 was 10 min. The reaction time for ST3Gal4, FUT7 and ST6Gal1 was 5 h. In each case, the labeling enzyme was subject to a 3-fold serial dilution starting from 1 μg each of B4GalT1, FUT7 and ST6Gal1 and 3 μg ST3Gal4. (**C**) Kinetic comparison of sLe^x^ synthesis by FUT6 and FUT7 from A2[3]. Reaction time was 20 min. Both FUT6 and FUT7 were subject to 6-fold serial dilution starting from 1 μg. (**D**) Kinetic comparison of Le^x^ synthesis by FUT6 and FUT9 from G2. Reaction time was 20 min. Both FUT6 and FUT9 were subject to 6-fold serial dilution starting from 1 μg.

As a further demonstration of glycan gel electrophoresis, we studied the enzymatic synthesis of Le^x^ and sLe^x^ using the antibody glycan G0 as a scaffold. For Le^x^ synthesis, G0 was first converted to G2 by B4GalT1 and then converted to G2F2 (carrier of Le^x^) by FUT6 or FUT9. For sLe^x^ synthesis, G0 was first converted to G2 by B4GalT1, then converted to A2[3] by ST3Gal4, and finally converted to A2[3]F2 (carrier of sLe^x^) by FUT7 (Figure 6A). During these steps, different amounts of enzymes and multiple reaction times were applied to reveal the intermediate products. Indeed, in Figure 6B, the intermediate products of G1, A1[3] and A1[6] during the synthesis of G2, A2[3] and A2[6], respectively, were observed. While it only took 10 min for the complete conversion of G0 to G2 by B4GalT1, it took 5 h for the complete conversion of G2 to A2[3] by ST3Gal4 and to A2[6] by ST6Gal1. No intermediate product was initially observed during the conversion of A2[3] to A2[3]F2 by FUT7 in a 5-h reaction. To search for the intermediate product, we performed additional experiments with FUT6 and FUT7 with only a 20-min reaction. Figure 6C shows the transition of A2[3] to A2[3]F2 by both FUT6 and FUT7, but with no distinct band for the intermediate, likely because the mobility shift caused by the modification is too small. Similar observation was made on the transition of G2 to G2F2 by FUT6 or FUT9 (Figure 6D).

Intermediate products are signs for the progress of each enzymatic step. Based on the amount of enzyme and the time needed for reaching the completion of each reaction, the relative velocities and therefore the activities of the enzymes in Figure 6 were estimated (Table I). It is noted that the FUTs have much faster kinetics than the sialyltransferases with the difference from one to three orders of magnitude. Considering that the FUTs are responsible for the final steps of Le^x^ and sLe^x^ formation, it is likely that these enzymes are subject to strict regulation, therefore allowing the cells to quickly respond to biological stimulation.

**Table I:** Reaction velocities based on reaction completion^a^ in Figure 6

Enzyme	Substrate	Product	Activity based on reaction completion (pmol/min/μg)^a^	Relative activity^b^
FUT6	A2[3]	sLe^x^	13.5	100
FUT6	G2	Le^x^	2.3	17
FUT7	A2[3]	sLe^x^	0.38	2.8
FUT9	G2	Le^x^	2.3	17
ST3Gal4	G2	A2[3]	0.0084	0.06
ST6Gal1	G2	A2[6]	0.025	0.19
B4GalT1	G0	G2	2.3	17

Note: ^a^The reaction velocity was calculated based on the time and amount of an enzyme required for the completion of a reaction but rather than the initial velocity used in Michaelis–Menten kinetics. Activities calculated based on completed reactions should be substantially lower than initial velocities but still give good estimations of overall real activities.

^b^Relative activities were normalized to that of FUT6 on sLe^x^ synthesis.

### Quantification of Cy5-labeled glycans by establishing a response curve for labeled G0 glycan standard

**Fig. 7:**
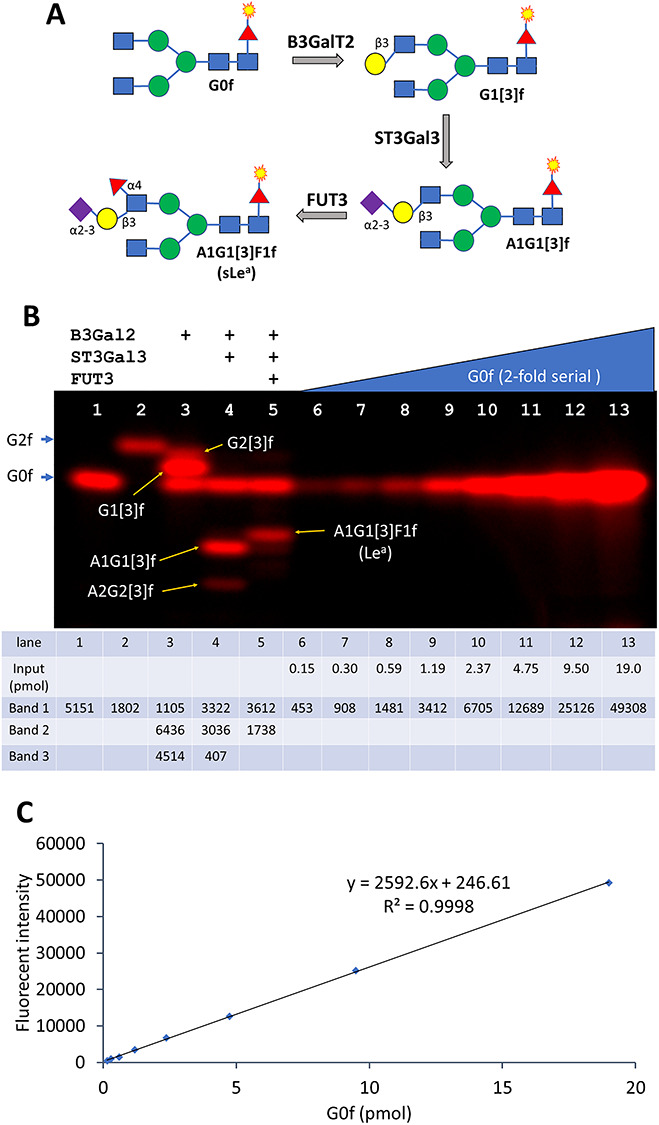
Quantification of sialyl Lewis a (sLe^a^) and its intermediate products. (**A**) scheme of the synthesis of sLe^a^ using B3GalT2, ST3Gal3 and FUT3. (**B**) The step-by-step synthesis of sLe^a^ and a 2-fold serial dilution of G0f. Lane 1 is labeled G0 (G0f) and lane 2 is labeled G2 (G2f). Lanes 3–5 contain the intermediate reactions of converting G0f to the final sLe^a^ carrying glycan A1G1[3]F1f with the indicated enzymes. Lanes 6–13 is a 2-fold serial dilution of G0f starting from 25 ng (19 pmol). The exposure time is 240 ms. The mass inputs of the serial dilution and all the band intensities were listed below the picture. (**C**) The response curve of the serial dilution in (**B**). The slope of the line represents the signal to mass ratio of G0f, which allows the quantification of the bands from lanes 1 to 5.

To evaluate the quantitative aspects of the glycan electrophoresis described in this article, we run a series of enzymatic reactions towards Le^a^ synthesis along with a 2-fold serial dilution of the FUT8-labeled antibody glycan G0 (G0f) (Figure 7). Le^a^ was synthesized from G0f sequentially via the steps of galactosylation by B3GalT2, sialylation by ST3Gal3 and fucosylation by FUT3 (Figure 7A). Major intermediate products of Le^a^ synthesis including G1[3]f, A1G1[3]f and A1G1[3]F1f were observed (Figure 7B). When the band intensities of the serial dilution of G0f in Figure 7B were plotted versus the masses of the glycan, a response curve with the linear coefficient of 0.9998 was obtained, with the slope of the curve representing the signal to mass ratio. The masses of the intermediates of Le^a^ synthesis were then calculated using the signal-to-mass ratio. While the reactions by ST3Gal3 and FUT3 were almost completed, B3GalT2 converted 53% and 9% of G0f to G1[3]f and G2[3]f, respectively. The results indicate that fluorophore-labeled glycans separated by electrophoresis can be quantitatively measured when a standard curve is established. In addition, the results also indicate that the sensitivity of the current method for glycan detection is below picomole level.

## Discussion

In this article, we demonstrated that various fluorophore-conjugated fucoses are well tolerated by FUTs. By incorporating these conjugated fucoses to target glycans and separating them through gel electrophoresis, we were able to reveal the substrate glycans as well as the substrate specificities of these FUTs. More specifically, we demonstrated the detection of specific N-glycans on therapeutic antibodies, RNase B and recombinant influenza viral neuraminidase. We also demonstrated step-by-step enzymatic synthesis of Le^x^ and sLe^x^ from defined glycan structure and further revealed that the responsible FUTs have kinetics one to three orders of magnitude faster than those of corresponding sialytransferases. Together with other glycosyltransferases and glycosidases, the methods allow quick detection of certain glycans and kinetic study of the biosynthesis of certain glycan epitopes. Our results support the notion that FUTs are subject to strict regulation for their roles in Le^x^ and sLe^x^ biosynthesis.

Electrophoresis is probably the most common technique for studying proteins and nucleic acids. In this report, we applied electrophoresis to study glycans. Mechanisms for glycan separation are based on the differences on charge, mass and molecular structures of the glycans in study (Wu et al. 2010). Charges can be introduced along with the incorporation of fluorophores that are usually negatively charged. Sialic acids naturally contribute negative charges; therefore, sialylated glycans have much faster mobility than other glycans. Neutral sugars such as galactose and fucose usually slow down the mobility of a glycan. Glycans with the same charge and mass maybe separated on structural differences. For examples, while monogalactosylated G1[3] and G1[6] were not separated in Figure 4B, di-sialylated A2[3] and A2[6] were well separated in Figure 4A. It would be ideal if a separated glycan on SDS–PAGE could be identified by comparison to a glycan standard. For this purpose, we have established a glycan ladder that contains six common antibody glycans in Figure 4A, which could be helpful for identifying glycans on IgG. For identifying unknown glycans, more glycan ladders will be required.

In comparison with protein electrophoresis, the current fluorophore-labeled glycan electrophoresis has the following advantages. First, glycans are covalently labeled, and the labeled molecules can be directly imaged under a fluorescent imager; therefore no staining step is required. Second, the labeled sample can be quantitated if a standard is provided. Third, after electrophoresis, gels can still be stained for proteins. By comparing the protein image and the fluorescent image of a same gel, the labeled glycans may be identified.

Glycan synthesis is not template driven, but rather determined by the availability of individual glycosyltransferases and their substrate glycans, therefore characterizing these glycosyltransferases and their kinetics is the key to the understanding of how glycan epitopes are synthesized. Our methods are especially suitable for studying these enzymatic processes, which is exemplified in the enzymatic synthesis of Le^x^ and sLe^x^ in Figure 6 and sLe^a^ in Figure 7.

## Material and methods

Recombinant FUT2, FUT3, FUT6, FUT8, FUT9, MGAT1, B3GalT2, B4GalT1, ST3Gal4, ST3Gal3, ST6Gal1, *Flavobacterium meningosepticum* PNGase F, *Streptococcus pyogenes* EndoS, Influenza viral H1N1 neuraminidase and GDP-Azido-Fucose were from Bio-Techne. Cantuzumab, an anti-Muc1 therapeutic antibody, was from Creative Biolabs. NIST monoclonal antibody reference material 8671 was from the National Institute of Standards and Technology. Alkyne Alexa Fluor® 488 and alkyne Alexa Fluor® 555 were from Thermo Fisher Scientific. IgG glycan G0 was from Agilent. Cy5-alkyne, RNase B, fetal bovine fetuin and asialofetuin and all other chemical reagents were from Sigma-Aldrich.

### Preparation of fluorophore-conjugated GDP-fucose

Fluorophore-conjugated GDP-fucoses were prepared by incubating equivalent GDP-Azido-Fucose (GDP-N_3_-Fuc) and alkyne-conjugated fluorophores via copper (I)-catalyzed azide–alkyne cycloaddition. For example, 5 mM of GDP-N_3_-Fuc was mixed with 5 mM of Cy5-alkyne in the presence of 0.1 mM of Cu^2+^ and 1 mM of ascorbic acid. The reaction was kept at room temperature for 2 h. The fluorophore-conjugated GDP-fucoses were purified on a HiTrap® Q HP (GE Healthcare) column and eluted with a 0–100% gradient of NaCl elution buffer (300 NaCl, 25 mM Tris at pH 7.5). The fluorophore-conjugated GDP-fucoses were collected based on color exhibition and UV absorption as the conjugated GDP-fucoses were vivid in color and had UV absorption at 260 nm. Alexa Fluor® 555 conjugated GDP-fucose, Alexa Fluor® 488 conjugated GDP-fucose and Cy5 conjugated GDP-fucose were prepared and purified and finally concentrated to >0.1 mM by a speed vacuum concentrator.

### Fluorescent labeling of glycoproteins and glycans and separation of labeled sample on SDS–PAGE

For a typical labeling reaction, 1–5 μg of a target protein was mixed with 0.2 nmol fluorophore-conjugated GDP-fucose and 0.2 μg of a fucosyltransferase in 30 μL 25 mM Tris pH 7.5, 10 mM MnCl_2_. The mixture was incubated at 37^o^C for 30 min. Longer incubation may increase labeling but not significantly (Supplemental Figure 3). To release labeled glycans from a glycoprotein, the sample was first denatured by heating at 95°C for 2 min in the presence of 0.5% SDS and 80 mM β-mercaptoethanol and then renatured with 1% Triton X-100 and finally treated with PNGase F at 10:1 mass ratio at 37°C for 20 min. To release glycans from an antibody, a sample was directly treated with Endo S at 10:1 mass ratio at 37°C for 20 min. All samples were separated by SDS–PAGE at 20 volts/cm. For separating labeled glycoproteins and antibodies, 4–20% gradient SDS gel was used. For separating free glycans, 15 or 17% gel was used. After separation, all gels were imaged using a FluorChem M imager (ProteinSimple, Bio-Techne). For glycoprotein samples, the gel was also imaged with traditional methods such as silver staining or trichloroethanol (TCE) staining.

### GlyQ analysis

All samples for GlyQ analysis were prepared and analyzed according to the manufacture’s protocol in Agilent Gly-Q™ Glycan Analysis System (formerly ProZyme).

## Author contribution

Z.L.W. designed and performed the experiments and wrote the manuscript. M.W. performed the GlyQ experiment. J.M.E. performed the experiment on Endo S releasing of glycans and labeling. A.D.P. and V.K. contributed to experimental design.

## Supplementary Material

Supplemental_Figures_cwaa030
