# Supplementary material for: Detecting substrate glycans of fucosyltransferases with fluorophore-conjugated fucose and methods for glycan electrophoresis
Source: Glycobiology. 2020 May 2;30(12):970–80. doi: 10.1093/glycob/cwaa030 (PMC7724747; doi:10.1093/glycob/cwaa030)
Supplement: Supplemental_Figures_cwaa030 [file supplemental_figures_cwaa030.pdf]

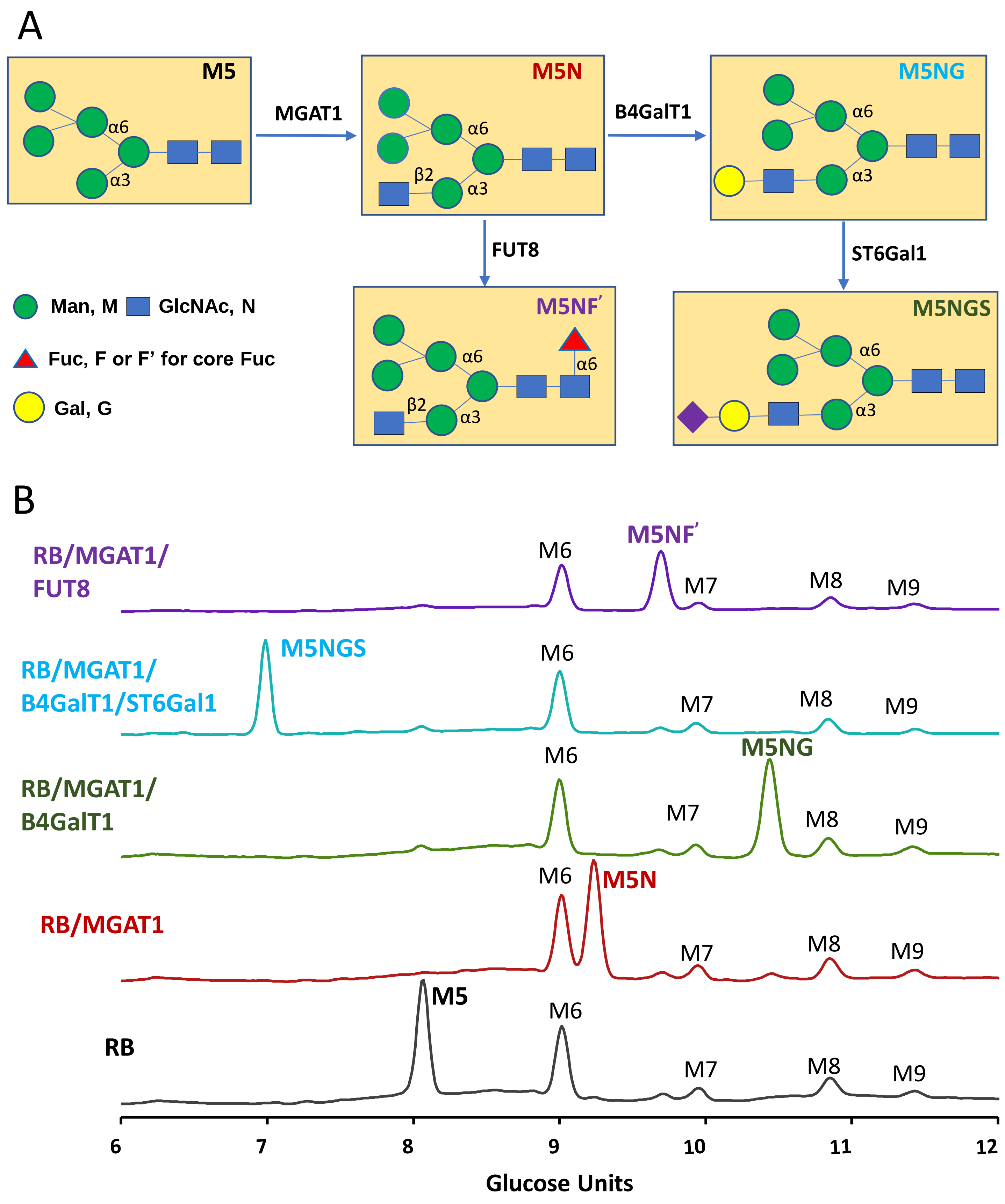

Supplemental Figure 1. Sequential conversion of Man5 by MGAT1, B4GalT1, ST6Gal1, and FUT8. A) The molecular structures of the glycans that were converted by the sequential enzymatic reactions in this study. In the nomenclature, M is mannose, N is GlcNAc, G is Gal, S is sialic acid, and F′ is core fucose. **B)** Showing the GlyQ analysis of sequential samples in Fig. 3B. The Man5 glycan (M5) of RNase B (RB) was the only glycan that was converted by these enzymes. It was sequentially converted by MGAT1 and B4GalT1 to M5N and M5NG, respectively. FUT8 was able to convert M5N to M5NF′. ST6Gal1 was able to convert M5NG to M5NGS.

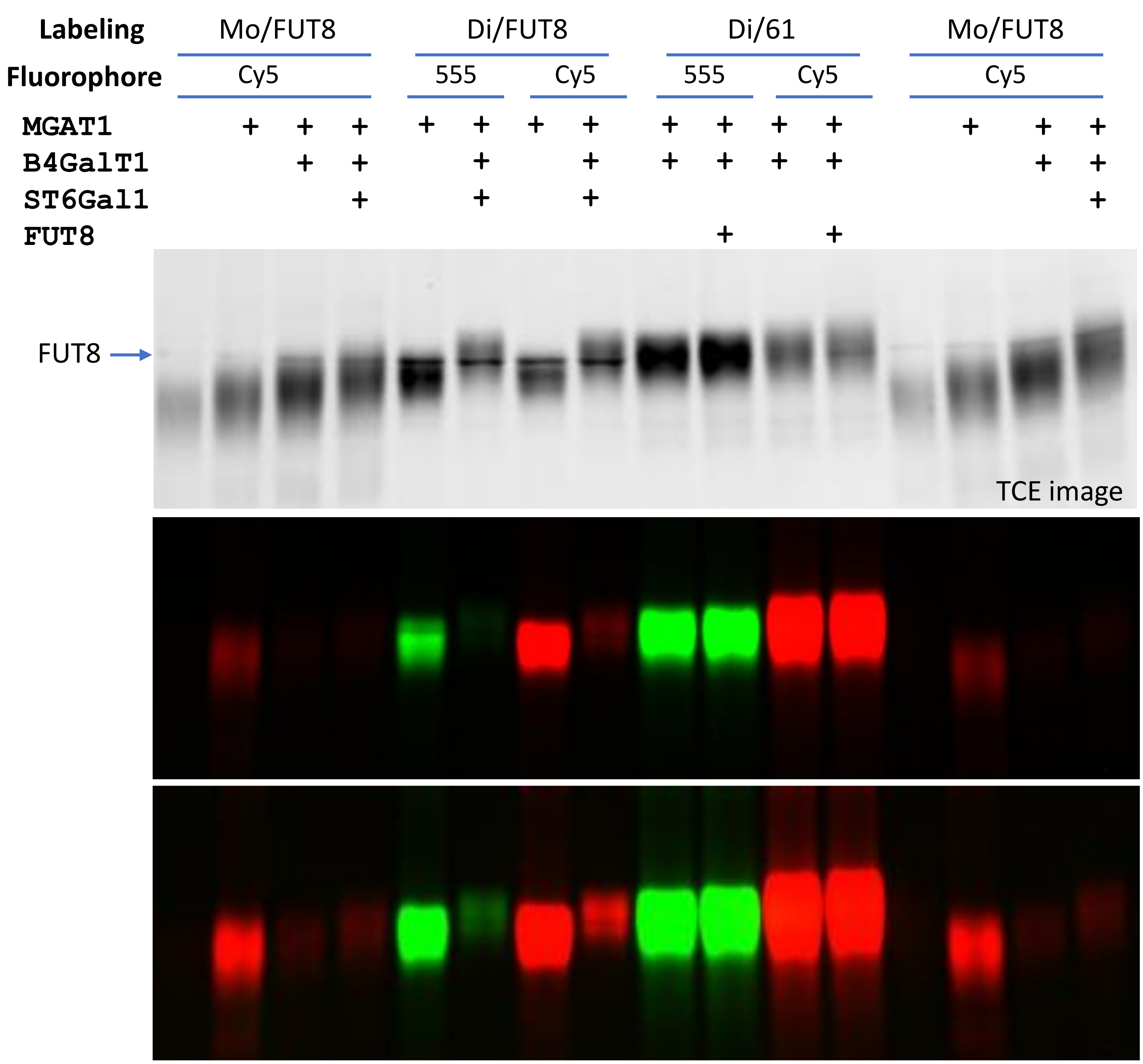

Supplemental Figure 2. The effect of sequential modification of Man3 on recombinant H1N1 influenza viral neuraminidase by MGAT1, B4GalT1, ST6Gal1 on FUT8 labeling. Recombinant 1918 H1N1 viral neuraminidase monomer (Mo) and dimer (Di) were used as substrates for the glycan modification. Pretreatment of the substrates for FUT8 labeling was performed by incubating the substrates with MGAT1, B4GalT1, and ST6Gal1 (in the presence of their natural donor substrates) for 30 minutes at 37°C. Labeling was achieved by incubating the pretreated samples with the labeling enzymes FUT8 and the fluorescent donor substrate GDP-AF555-Fuc (555) or GDP-Cy5-Fuc (Cy5). For comparison, the dimeric neuraminidase was also pretreated with MGAT1 and FUT8 (in the presence of their natural donor substrates) at 37°C for 30 minutes, and then labeled by incubating with B4GalT1 (B4) and ST6Gal1 (61) in the presence of the fluorescent donor substrates of CMP-AF555-SA or CMP-Cy5-SA. The middle and lower panels are fluorescent images with different contrast.

This experiment demonstrate the following:

1. The unmodified α1-3 arm GlcNAc residue introduced by MGAT1 is most critical for FUT8 recognition.
2. Extension of the α1-3 arm GlcNAc residue by B4Gal1 and ST6Ga1 significantly inhibit FUT8 recognition.
3. While extension of the α1-3 arm GlcNAc residue by B4GalT1 and ST6Gal1 inhibits core-6 fucosylation, core-6 fucosylation has no obvious effect on ST6Gal1 substrate recognition.

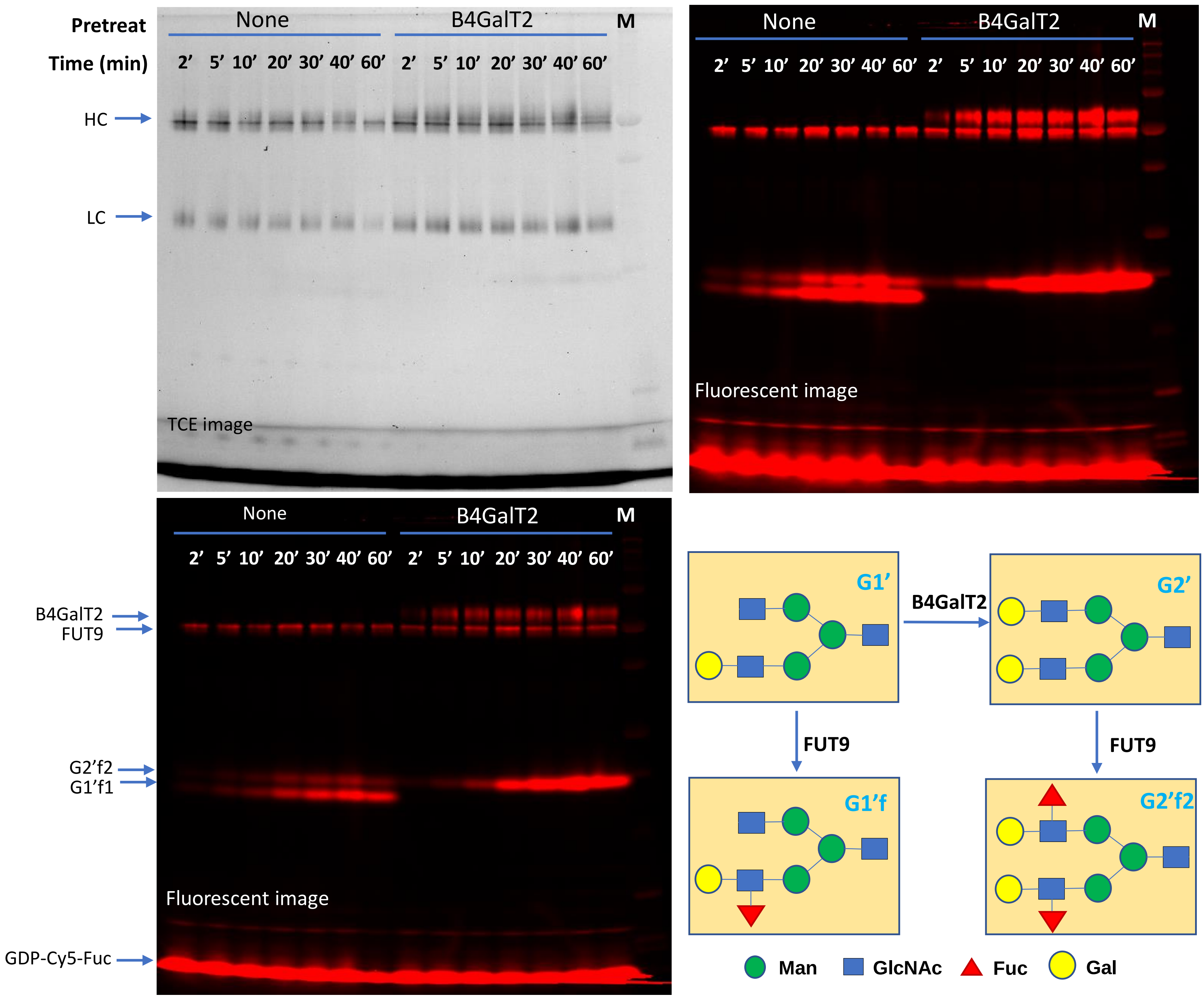

Supplemental Figure 3. Kinetic study of the labeling by FUT9. Mouse IgG (Bio-techne, R&D Systems) was digested with Endo S thoroughly and labeled with 0.3 µg of FUT9 and 0.2 nmol of GDP-Cy5-Fuc in 30 µl at 37° C for the indicated time points. The glycans were also pretreated with B4GalT2 for 5 minutes at 37° C and then labeled in the same way. The reactions were separated on 15% SDS PAGE. B4GalT2 pretreatment converted all G1’ to G2’. Maximal labeling on G1’ and G2’ were achieved around 30 minutes. FUT9 showed self-labeling. B4GalT2 was labeled as well and maximal labeling was achieved at 20 minutes. The heavy chain (HC) and light chain (LC) were visible in the TCE image. M, Western blot marker (Bio-Rad).
